# Mapping potential cultural and religious tensions in end-of-life care for Muslim patients: A scoping review

**DOI:** 10.1177/02692163251407877

**Published:** 2026-01-21

**Authors:** George Muishout, Lenneke Post, Salima El Ayachi, Johannes C. F. Ket, Lia van Zuylen

**Affiliations:** 1 Department of Spiritual Care, Amsterdam UMC, The Netherlands; 2 Cancer Treatment and Quality of Life, Cancer Center Amsterdam, The Netherlands; 3 School of Religion and Theology, Faculty of Social Sciences and Humanities, Vrije Universiteit Amsterdam, The Netherlands; 4 Medical Library, Vrije Universiteit Amsterdam, The Netherlands; 5 Department of Medical Oncology, Amsterdam UMC, The Netherlands

**Keywords:** end-of-life-care, Muslim patients, ethical dilemmas, cultural dilemmas, religious dilemmas

## Abstract

**Introduction:**

Muslims constitute the largest religious minority in Euro-American countries. Interactions between Muslim patients and healthcare providers in end-of-life care may give rise to religio-cultural ethical dilemmas, resulting in tensions. Understanding these tensions may support care providers in navigating the critical transition from life to death within non-Muslim majority contexts.

**Aim:**

To thematically map ethical tensions arising from religio-cultural dilemmas in end-of-life care for Muslim patients, and to equip healthcare professionals in Euro-American contexts with the insights needed to address them.

**Design:**

A scoping review was conducted in accordance with the Johanna Briggs Institute (JBI) methodology.

**Data sources:**

A comprehensive literature search was performed in Scopus, Medline, CINAHL, and APA PsycInfo, from inception to 7 November 2024, in collaboration with a medical information specialist. Empirical studies addressing religio-cultural ethical dilemmas and tensions in end-of-life care for Muslims were included.

**Analysis:**

Ethical tensions were examined using a structured framework integrating theological perspectives with empirical findings.

**Results:**

Of 9237 records screened, 12 studies met the inclusion criteria. Three overarching themes emerged: (1) end-of-life communication, (2) spiritualizing pain, and (3) treatment withholding/withdrawal. Tensions were most pronounced when divergent moral frameworks prioritized conflicting principles.

**Conclusions:**

Ethical tensions in end-of-life care for Muslim patients in Euro-American healthcare settings reflect diverse, context-dependent interpretations of Islamic principles. Addressing these tensions requires early, culturally sensitive engagement and the development of a shared ethical framework in close collaboration with healthcare providers, Muslim chaplains, mosques, imams, and umbrella organizations.

**What is already known about the topic**

- Euro-American healthcare increasingly engages with Muslim patients and families.
- Differences between biomedical and Islamic medical ethics may complicate end-of-life care.
- Limited cultural humility, responsiveness, and safety can lead to deadlocks between healthcare providers and Muslim patients and/or their families in end-of-life care.

**What this paper adds**

- A comprehensive ethical analysis by mapping key ethical tensions through a structured ethical framework, while integrating theological insights with empirical findings.
- Religion shapes end-of-life care through both lived experience and normative Islamic theology.
- Islamic perspectives are diverse, offering interpretive flexibility that can help bridge ethical divides.

**Implications for practice, theory or policy**

- Individually engaged culturally attuned communication, using language aligned with religious sensibilities, can foster mutual understanding.
- Collaboration with Muslim communities, including mosques and grassroots organizations, can raise awareness about end-of-life care and develop shared care frameworks.
- Muslim chaplains within hospitals can mediate perspectives and support culturally sensitive care.

## Introduction

Muslims are the world’s largest religious minority in Euro-American countries. They are projected to account for 10% of Europe’s population and 2.1% of the U.S. population by 2050.^1^ A natural consequence will be an increase in intercultural interactions within Euro-American clinical practice between care providers and Muslim patients and their families. This increase becomes particularly relevant during end-of-life care, where a difference in ethical principles may give rise to tensions regarding the definition of what constitutes good care for Muslim patients.^2^

While, broadly speaking, common medical ethics in Euro-American clinical contexts emphasize human reason and individual patient rights, Islamic medical ethics are a category of applied theology rooted in the Quran and the sayings of the Prophet Muhammad, transformed by specialized Islamic scholars into practical ethical advice for lay Muslims in specific clinical situations, including end-of-life care.^3^ Alongside this scholarly discourse exists the domain of ‘lived religion’, which examines how Muslims embody, practice, and experience their faith in everyday life (i.e. what people do with their religion).^4^ In practice (and research), it is often challenging to disentangle culturally and religiously informed action orientations from the choices Muslims make in end-of-life care. Therefore, in this study, the term ‘religio-cultural’ is employed as a compound noun to encompass both dimensions, capturing the mutual influence and dynamic interplay between religion and culture, as well as between applied theology and lived religion.^4^ Specific themes of religio-cultural ethical dilemmas that may arise in the care of Muslim-patients include truth telling regarding impending death, the withholding or withdrawal of life-sustaining treatment, and the use of continuous palliative sedation.^5–8^ The resulting deadlocks may create professional uncertainty, stemming from a lack of cultural knowledge and/or the necessary cultural humility and responsiveness to address these deadlocks more effectively.^9^ These tensions are further complicated by the diversity of religious interpretations within Islamic ethics, which themselves are subject to cultural change over time and across contexts.^10^ In Muslim-majority settings, religious interpretations—whether derived from applied theology or shaped by lived religion—may culturally reinforce practices such as withholding information from dying patients. However, when these practices are transferred to non-Muslim majority healthcare systems—where transparency and individual autonomy are emphasized—they may give rise to ethical tensions that result in professional uncertainty.

In recent years, a limited number of review studies have emerged highlighting some of the challenges and dynamics of providing care for Muslims in end-of-life settings.^11–15^ What is lacking in the current review literature is a study that specifically outlines the tensions resulting from religio-cultural ethical dilemmas that may arise in end-of-life care for Muslim patients. To address this issue, the current review aims to thematically map these tensions and dilemmas, identify gaps in what is known, and potentially equip healthcare providers with the knowledge and insights needed to approach potential deadlocks.

## Methods

A scoping review was selected as the methodological approach, as it is well-suited for providing a comprehensive overview of existing studies, while also enabling the identification of gaps within the current body of research literature.^16^ The review was conducted in accordance with the Joanna Briggs Institute (JBI) methodology for scoping reviews, which emphasizes systematic searching, transparent selection, and iterative data charting using a PCC (Population, Concept, Context) framework.^17^ The review was executed by a multidisciplinary research team with expertise in systematic review methodology and search strategies (JCFK), Islamic ethics (GM, SEA), religious studies (GM, LP), and clinical oncology and palliative care (LVZ, SEA, LP). This diverse composition enhanced the methodological rigor and ensured that the interpretation of findings was informed by theological, cultural, and clinical perspectives.

The primary question explored in this scoping review was: What are the key religio-cultural ethical dilemmas that may lead to tensions during the process of end-of-life care for Muslim patients and their families in interactions with care providers in non-Muslim majority healthcare systems? A secondary question—What is known about recommendations for care providers aimed at early identification and mitigation of tensions in end-of-life care with Muslim patients and their families—was initially included in the scope of this review. However, since none of the search results specifically addressed recommendations or best practices for approaching or mitigating religio-cultural ethical tensions in a non-Muslim majority healthcare system context, this question was excluded from further analysis.

In this review, the term ‘tension’ is understood as the consequence of religio-cultural ethical dilemmas and conflicts that arise when the values, beliefs, and expectations of Muslim patients and/or their families—informed by normative Islamic scholarship and shaped by lived religion (i.e. how individuals interpret and enact their faith in everyday life)—come into conflict with the clinical, biomedical, ethical, and/or communicative frameworks of their healthcare providers. These tensions may manifest as disagreement, practical dilemmas in clinical decision-making, emotional and existential distress, or a combination thereof. They typically arise when differing ethical frameworks prioritize conflicting moral principles. In the following we will use the term ‘tensions’ to describe these dynamics.

## Eligibility criteria

Empirical studies were included that described tensions in end-of-life care for Muslim patients. These encompassed attitude studies, descriptive and prescriptive analyses, case-based approaches, and hypothetical scenarios. Studies were included regardless of publication year but limited to peer-reviewed articles published in English. Both qualitative and quantitative methodologies were considered. A key inclusion criterion was the presence of tensions caused by conflicting interpretations of clinical biomedical and Islamic medical ethics—for example, autonomy versus divine-based beneficence. Consequently, studies included were mostly studies from Europe and North America, where intercultural dynamics in clinical settings can be conflictual. Moreover, we included studies from Muslim-majority countries (e.g. Saudi Arabia and Sudan), where divergent interpretations of biomedical and Islamic medical ethics may also emerge. Studies were excluded if they were non-empirical, focused solely on normative Islamic perspectives, or if religion and culture were absent from the results. To enhance clarity and accessibility, the eligibility criteria are presented in tabular format:

## Information sources and search strategy

A comprehensive search was performed in the databases: Elsevier/Scopus, OVID/Medline, Ebsco/CINAHL, and Ebsco/APA PsycINFO from inception to November 7, 2024 in collaboration with a medical information specialist (JCFK). The search included controlled and free text terms for synonyms of ‘end of life’ or ‘terminal care’ and ‘Islam’. The search was performed without restrictions for methodology or date. The full search strategies can be found in the Supplemental Materials. Duplicate articles were excluded by JCFK using Endnote X20.0.1 (Clarivatetm), following the Amsterdam Efficient Deduplication (AED)-method and the Bramer-method.^18,19^ Mendeley was used to organize the collected data, ensuring a systematic approach.^20^ To ensure reporting rigor the Prisma Extension for Scoping Reviews (PRISMA-ScR) was used as a guideline to report the results.^18^ The project has been officially registered as a protocol on the Open Science Framework (OSF) and can be accessed using the following DOI:https://doi.org/10.17605/OSF.IO/S364M. The scoping search was carried out on November 7th 2024 without any date restrictions to ensure a comprehensive overview.

## Study selection

The search results including the abstracts were imported into Rayyan, a web app for systematic reviews, for further analysis and organization.^21^ All abstracts were initially screened by GM and LP with the blinding function enabled to prevent bias. Data that were unclear, such as those lacking an abstract, were labeled under the category ‘maybe’, with marked records flagged for further discussion. The full text was subsequently downloaded, imported into Rayyan, and reviewed according to the eligibility criteria. Variations in the interpretation of data inclusion and exclusion were resolved through discussions with SEA, leading to a consensus on the final interpretation. The next stage of the screening process involved full-text reviews of studies categorized as ‘maybe’ or ‘included’, organized within Rayyan for further analysis, based on the inclusion and exclusion criteria as detailed under the next subsection.

## Data extraction

A data charting form was developed based on the initial research questions and eligibility criteria, with the aim of mapping key tensions and, where available, recommendations in end-of-life care. The charting form was iteratively refined during the review to better align with the study objectives. Data items extracted included: study objectives, country of origin, study design and methodology, participant type, and key findings (nature of the tension, religious and cultural context, clinical implications). Studies using the same dataset were combined to avoid duplication. The extraction procedure was guided by a protocol accessible via cloud storage to GM, LP, and SEA. GM and LP conducted the thematic organization of the data, using the blind feature in Rayyan to minimize bias. Discrepancies were resolved through discussion between GM and LP, and in consultation with SEA when needed. Following the screening phase in Rayyan, Excel was used to compile a final overview table of the included studies.

## Data analysis and synthesis

In line with JBI guidance on qualitative synthesis, the analysis and presentation of data involved a structured interpretative process. GM and LP reviewed the results and prepared the final data chart. A PRISMA flow diagram was used to visualize the screening process, including inclusion and exclusion of studies. Excel was used to organize and refine the characteristics of the final data chart. In shaping the analytical framework, particular attention was paid to how tensions arise from conflicting interpretations of clinical biomedical principles and religio-cultural Islamic medical ethics. To structure the analysis, a dual approach was applied. First, tensions were identified inductively from empirical descriptions of dilemmas in end-of-life care, shaped by the interplay of religious norms and culturally embedded expectations, with clinical biomedical practice. In this process, particular attention was paid to the tension between religion and culture—especially where religious norms are interpreted and enacted differently across contexts. This distinction supported the analysis of how ethical reasoning may be shaped not only by theological sources but also by culturally embedded expectations. These tensions were interpreted through a deductive lens using a comparative ethical framework. Specifically, they were mapped onto Beauchamp and Childress’s four-principle model—autonomy, beneficence, nonmaleficence, and justice—and contrasted with Islamic equivalents as formulated by Chamsi-Pasha and Albar. These include divine-based family decision-making as an alternative to autonomy, divine-based beneficence, divine-based nonmaleficence, and divine-based justice, reflecting a theological and communal orientation in Islamic medical ethics.^3^ Table 1 provides a comparative overview of these principles. This dual approach enabled conceptual categorization of tensions based on differing prioritizations and interpretations of general biomedical and Islamic medical ethics. The selection of this comparative framework is supported by the combined expertise of its authors in clinical medicine and Islamic jurisprudence. Albar, in particular, is recognized by many Muslim jurists as a credible authority in Islamic medical ethics, with a background that bridges clinical practice and normative Islamic reasoning.^22^ We also acknowledge that the Beauchamp and Childress principles are not interpreted uniformly across Euro-American healthcare systems. For example, while autonomy and informed consent are central in many contexts, practices such as euthanasia are legally permitted in some countries and considered morally impermissible in others. This underscores that both general and Islamic ethical principles are contextually enacted and subject to varying normative evaluations. Throughout the data analysis, GM and LP conducted weekly evaluations via online meetings. The final results were discussed both on- and offline with all members of the research team, leading to the refinement and finalization of the outcomes.

**Table 1.** Features of biomedical versus Islamic medical ethics.

Principle	Biomedical ethics (self-determination-based)	Islamic equivalent or alternative Islamic principle	Islamic medical ethics (divine-based)
Beneficence	Doing good for the patient, guided by human reason and experience	Divine-based beneficence	Faith-based duty to do good, fully endorsed by Islamic teachings and religious obligations
Patient Autonomy	Emphasizes individual rights and self-determination	Divine-based family based decision-making	Emphasizes family decision-making, with responsibility toward God and guided by religious duties toward God and others
Nonmaleficence	Avoiding harm, based on clinical judgment	Divine-based nonmaleficence	Prioritizes avoiding harm over bringing good, based on clinical judgment, endorsed by Islamic jurisprudence and Islamic teachings.
Justice	Fair distribution of medical resources, based on principles of equity and fairness	Divine-based justice	Moral obligation and command of God, focusing on fairness and equity as defined by religious teachings

Source: Adapted from Chamsi-Pasha and Albar.^3^

Distinctive features:

* Islamic Medical Ethics: Divine-based, applied by Islamic scholars to provide practical ethical advice.

* Biomedical Ethical Approach: Based on human reason and experience, emphasizing individual rights and autonomy.

## Results

**Figure 1.**
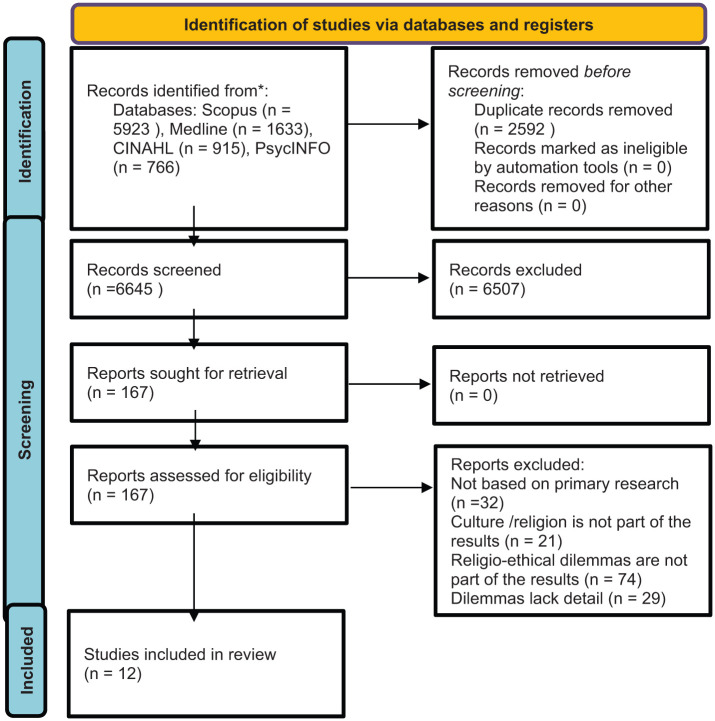
Prisma flow chart of scoping review process.

From the initial pool of 9237 search results, 6645 records were screened for eligibility after duplicate removal. Subsequently, a full-text review of 167 studies was performed, leading to the inclusion of 12 studies in the final scoping review. The associated PRISMA flowchart provides a detailed overview of the review process (Figure 1).

## Study characteristics

Of the 12 studies included, 10 were conducted in Euro-American countries. These include the Netherlands (*n* = 4), the United Kingdom (*n* = 1), the United States (*n* = 2), Belgium (*n* = 1), Scotland (*n* = 1), and Sweden (*n* = 1).^23–32^ Two of the Dutch studies were based on the same dataset.^23,25^ The remaining studies were conducted in Muslim-majority countries: Saudi Arabia (*n* = 1) and Sudan (*n* = 1)^33,34^ Eleven studies employed qualitative designs, using interviews (*n* = 8) and focus groups (*n* = 2)^23–30,32,33^ One study was quantitative, using a questionnaire to assess attitudes toward euthanasia among Muslim medical students in Sudan (*n* = 152).^34^ The qualitative studies included Muslim patients and relatives, healthcare professionals (both Muslim and non-Muslim), and Muslim religious leaders. Sample sizes in these qualitative studies ranged from small targeted groups (*n* = 10) to larger multi-perspective studies (up to *n* = 76), with a total of 327 participants across all qualitative studies (excluding duplicate datasets). Including the quantitative study, the combined total sample size across all studies was 479. For detailed study characteristics, see Table 2.

**Table 2.** Characteristics included studies.

Authors	Title	Study objectives	Country of origin/setting	Type of study	Method(s)	Participants	Key findings
Abudari, G., Hazeim, H., & Ginete, G. (2016).	Caring for terminally ill Muslim patients: Lived experiences of non-Muslim nurses.	Examining non-Muslim nurses’ experiences in caring for terminally ill Muslim patients and their families.	Saudi Arabia. Palliative care unit of a tertiary care hospital with a cancer center in Riyad.	Qualitative	Semi-structured interviews, interpretative phenomenology.	Non-Muslim nurses with at least 2 years’ experience of caring for terminally ill Muslim patients, and being able to communicate in English (*n* = 10)	Nurses’ experiences were shaped by family matters, end-of-life practices, and challenges. Cultural values, religious practices, and a family-centered approach influenced care. Issues like the absence of palliative care integration and lack of interdisciplinary team members affected their work. Additionally, nurses lacked cultural knowledge due to insufficient awareness and formal education.
Ahmed, A. M., & Kheir, M. M. (2006).	Attitudes toward euthanasia among final-year Khartoum University medical students	Exploring the perspectives of final-year medical students at Khartoum University on euthanasia.	Sudan. Faculty of medicine, Khartoum.	Quantitative	Questionaire	Muslim final year medical students (*N* = 152; response rate: 83.5%)	Most medical students (76.6%) opposed euthanasia, citing religious and ethical concerns, while 23.4% supported it to alleviate suffering and respect autonomy. Strongly religious students were more likely to oppose (95.4%) than moderately religious ones (46.3%)
Duffy, S. A., Jackson, F. C., Schim, S. M., Ronis, D. L., & Fowler, K. E. (2006).	Racial/ethnic preferences, sex preferences, and perceived discrimination related to end-of-life care.	Obtaining in-depth information on end-of-life preferences across five racial/ethnic groups in Michigan	United States, professional marketing facility, Islamic center (Arab Muslim women’s group).	Qualitative	Focus groups and follow-up survey	Arab Muslims, Arab Christians, Hispanics, blacks, and whites (*n* = 73)	End-of-life preferences vary across groups, highlighting the need for culturally sensitive care as diversity grows.
Kolmar, A., Kamal, A. H., & Steinhauser, K. E. (2022).	Clinician end-of-life experiences with pediatric Muslim patients at a US quaternary care center.	Addressing the unique dynamics of pediatric clinician experiences with Muslim patients and their families in the EOL setting	United States	Qualitative	Semi-structured interviews, thematic analysis	Clinicians (*n* = 16; 5 physicians, 5 social workers, and 6 nurses), the majority of whom were female, Caucasian, and Christian in an institution where Muslim patients are a significant minority.	Many clinicians recognize they likely provide disparate care to minority patients for a variety of reasons such as language barriers, difficulty engaging with Muslim families, variations in approach to care and communication, discomfort with gender roles, moral distress with unrelatable decision-making, and external pressures on patient decision-making.
Lundqvist, A., Nilstun, T., & Dykes, A. K. (2003).	Neonatal End-of-Life Care in Sweden: the views of Muslim Women	Exploring Muslim women’s views of neonatal end-of-life-care in Sweden.	Sweden.	Qualitative	Interviews using a standardized questionnaire with open-ended questions, content analysis.	Muslim mothers (*n* = 11)	Key themes identified include useful yet threatening information, maternal emotions, others’ roles, protecting the infant, grief from memories, traditions, and belief in life after death
Muishout, G., Topcu, N., de la Croix, A., Wiegers, G., & van Laarhoven, H. W. (2022).	Turkish imams and their role in decision-making in palliative care: A Directed Content and Narrative analysis.	Gaining knowledge about how imams shape their roles in decision-making in palliative care.	The Netherlands.	Qualitative	Semi-structured interviews, direct content analysis, narrative analysis.	Turkish imams with proficiency in supporting congregants during palliative care (*n* = 10)	Imams frequently advised against consenting to the withdrawal or termination of medical treatment, as they believe it contradicts faith in God’s omnipotence and could hold all involved Muslims accountable in the afterlife. Fatwas from expert Muslim committees significantly shape medical decision-making in palliative care
Muishout, G., de la Croix, A., Wiegers, G., & van Laarhoven, H. W. (2022).	Muslim doctors and decision making in palliative care: A discourse analysis.	Exploring how Muslim doctors shape their attitudes and approaches to palliative decision-making.	The Netherlands.	Qualitative	Semi-structured interviews,discourse analysis, word frequency analysis.	Muslim doctors with experience with administering palliative sedation (*n* = 10) Same participant group used in both the 2018 and 2022 studies by Muishout et al.	Six discourses, with the avoidance of suffering as standard medical care emerging as dominant were identified. Muslim doctors’ attitudes toward palliative decision-making were primarily shaped by the standardized goal of preventing suffering, rather than religious motivations.
Muishout, G., van Laarhoven, H. W. M., Wiegers, G., & Popp-Baier, U. (2018).	Muslim physicians and palliative care: attitudes toward the use of palliative sedation.	To understand the professional experiences of Muslim physicians with palliative sedation, considering both religious and professional norms	The Netherlands.	Qualitative	Semi-structured interviews, Interpretative phenomenology		Participants stressed their duty to make treatment decisions, even if these conflicted with Islamic scholars’ views. Most felt morally obligated to alleviate patients’ pain in the final stage of life, considering the absence of death acceleration essential for palliative sedation
Oosterveld-Vlug, M. G., Francke, A. L., Pasman, H. R. W., & Onwuteaka-Philipsen, B. D. (2017).	How should realism and hope be combined in physician-patient communication at the end of life? An online focus-group study among participants with and without a Muslim background.	Examining perspectives on the optimal combination of realistic and hopeful information in physician–patient communication at the end of life.	The Netherlands.	Qualitative	Online focus groups, thematical analysis.	Three separate groups: (1) non-Muslim patients, older people, and relatives (*N* = 24, 2 groups), (2) non-Muslim healthcare professionals (*N* = 21, 2 groups), (3) Muslim patients and relatives (*N* = 9, 1 group).	Participants preferred that doctor’s should empathetically provide realistic information, support the shift from ‘hope for a cure’ to ‘hope for a good death’, and involve care teams. Muslims prefer information through relatives and avoiding terms like ‘incurable illness’ opting instead for a focus on the absence of further curative options.
Suleman M. (2023).	The balancing of virtues-Muslim perspectives on palliative and end of life care: Empirical research analyzing the perspectives of service users and providers.	Enhancing current narratives on palliative and end-of-life care by exploring how faith values and virtues of Muslim patients and families are experienced.	United Kingdom	qualitative	Semi-structured interviews, thematical analysis	Muslim patients & families, healthcare professionals, chaplains, and community faith leaders (*n* = 76)	Navigating the intersection of diverse values and moral frameworks requires healthcare providers to engage in nuanced and thoughtful negotiation when supporting Muslim patients and families in end-of-life care.
Van den Branden, S., & Broeckaert, B. (2008).	Medication and God at interplay: End-of-life decision-making in practicing male Moroccan migrants living in Antwerp, Flanders, Belgium.	Exploring how elderly Moroccan men in Antwerp (Flanders, Belgium) connect their faith in God to their use of and experiences with medication in end-of-life situations.	Belgium, Antwerp	Essay based on self conducted qualitative research	Semi-structured in depth interviews	Maroccan men (*n* = 10), leading Antwerp imams (*n* = 2), Muslim general practitioners (*n* = 2)	Religious unacceptability of euthanasia. Shared belief that God determines lifespan, guiding the pursuit of medical care within divine limits. Doctors and imams agree that while God provides cures, humans must use intellect to uncover and apply them. Imams emphasize God’s absolute omnipotence more strongly than physicians.
Worth, A., Irshad, T., Bhopal, R., Brown, D., Lawton, J., Grant, E., Murray, S., Kendall, M., Adam, J., Gardee, R., & Sheikh, A. (2009).	Vulnerability and access to care for South Asian Sikh and Muslim patients with life limiting illness in Scotland: prospective longitudinal qualitative study	To understand the care experiences and access challenges faced by South Asian Sikh and Muslim patients with life-limiting illnesses in Scotland, and explore solutions.	Scotland (central)	qualitative	A longitudinal, prospective approach using in-depth interviews.	Purposively selected South Asian Sikh and Muslim patients (*n* = 25), family carers (*n* = 18), key health professionals (*n* = 20).	Services often fail to provide culturally appropriate end-of-life care, with barriers including resource limitations, discrimination, lack of awareness about hospices, and challenges discussing death. Vulnerable groups like recent migrants, non-English speakers, those without family advocates, and patients with non-malignant diseases face the greatest care inadequacies.

## Major themes and ethical dilemmas

We identified three major themes where ethical differences resulted in tensions, including *(1) End of life communication; (2) Spiritualizing pain; (3) Treatment Withholding & Withdrawal.* Studies were labeled as encompassing prior experienced, as well as potential future tensions, depending on the study type. Within the themes, all studies included were individually detailed in terms of conflicting principles that evoke ethical dilemmas and tensions within the triadic relationship between the care provider, the Muslim patient, and their family. In religiously inspired ethical principles the term ‘Divine-based’ was used, followed by the specific principle (Beneficence, Patient Autonomy, etc.) as shown in Table 3. Consequently, key issues were described, and clinical implications were indicated. See Table 4.

**Table 3.** Eligibility criteria.

Criterion	Specification
Study type	Empirical studies, including attitude studies, descriptive/prescriptive analyses, case-based approaches concerning prior experienced or potential future tensions.
Language	English only.
Publication status	Peer-reviewed publications.
Date range	No restrictions on publication year.
Methodology	Both qualitative and quantitative studies.
Geographical scope	Studies from European, American and Muslim-majority countries.
Rationale for scope	To explore religio/cultural ethical tensions between biomedical and Islamic frameworks.
Inclusion criteria	Studies must describe tensions because of conflicting interpretations of biomedical and Islamic medical ethics.
Exclusion criteria	Non-empirical studies; studies focusing solely on normative Islamic perspectives without addressing tensions; studies where religion/culture are absent from results.

**Table 4.** Major themes, dilemmas, and implications.

Included studies	Key issue	Ethical dilemma (As perceived by (APB))	Description & clinical implications
End of life communication			
Oosterveld-Vlug, M. G., Francke, A. L., Pasman, H. R. W., & Onwuteaka-Philipsen, B. D. (2017). (potential future tension)	Family control over end-of-life communication	Divine based non-maleficence and /or divine based beneficence (family) versus Patient Autonomy (care providers; APB Muslim participants)	Muslims prefer that physicians first provide realistic information to the patient’s relatives to protect patients (Divine-based non maleficence) Subsequently, open and explicit communication with the patient does not always occur. They advise doctors to inform the patient and/or relatives about the lack of curative treatments, but avoid stating the illness is incurable, as life and death decisions are beyond their authority (divine based beneficence), that may hinder care providers from offering full disclosure to the patient (Patient Autonomy).
Duffy, S. A., Jackson, F. C., Schim, S. M., Ronis, D. L., & Fowler, K. E. (2006).(potential future tension)	Truth telling versus truth veiling	Divine based non-maleficence (family) versus Patient Autonomy (caregivers; APB Arab Muslims & Christians)	To protect patients, Arab (Muslim and Christian) families often avoid discussing terms like death or cancer with dying patients, choosing partial truths instead (divine based non-maleficence), though many prefer full disclosure for themselves to prepare for the afterlife. This practice may compromise the patient’s right to know (patient autonomy).
Worth, A., Irshad, T., Bhopal, R., Brown, D., Lawton, J., Grant, E., Murray, S., Kendall, M., Adam, J., Gardee, R., & Sheikh, A. (2009). (prior experienced tension)	Open dialog about death	Divine based non-maleficence (family) versus Patient Autonomy and non-maleficence, beneficence (APB caregivers)	Families, frequently serving as interpreters, may withhold information to maintain the patient’s hope (divine based non maleficence), leading to conflicts with healthcare providers who value transparency (patient autonomy). The absence of open dialog about dying may complicate planning effective end-of-life care (non-maleficence, beneficence).
Abudari, G., Hazeim, H., & Ginete, G. (2016). (prior experienced tension)	Family control over end-of-life communication	Divine based non-maleficence and/or family based decision-making (family) versus Patient Autonomy, non-maleficence, beneficence (caregivers; APB non-Muslim nurses)	To protect patients, families manage end-of-life decisions and withhold information (divine based non maleficence, divine based, family based decision- making potentially affecting patient autonomy and care quality.
Spiritualizing pain			
Muishout, G., de la Croix, A., Wiegers, G., & van Laarhoven, H. W. (2022). & Muishout, G., van Laarhoven, H. W. M., Wiegers, G., & Popp-Baier, U. (2018). (prior experienced tension)	Pain management	Divine based beneficence (family) versus non maleficence (caregivers; APB Muslim doctors)	A (Muslim) doctor’s duty to relieve pain (non-maleficence) may conflict with beliefs that promote enduring pain as a means of purification, advocated by family members on behalf of patients unable to express their wishes (divine based beneficence).
Van den Branden, S., & Broeckaert, B. (2008). (potential future tension)	Pain management	Divine based beneficence (family) versus non-maleficence (caregivers; APB by a single Muslim participant)	Pain medication with a double effect may be rejected, as enduring pain is viewed by some as a means of redemption and a path to forgiveness in the afterlife (divine based beneficence), potentially conflicting with the right to pain relief (non-maleficence).
Ahmed, A. M., & Kheir, M. M. (2006). (potential future tension)	Euthanasia	Divine based non-maleficence (family) versus patient Autonomy (APB Muslim university medical students)	Respondents view euthanasia as forbidden due to religious beliefs, concerns about misuse, and its potential to obstruct end-of-life research (divine based non maleficence). Conversely, it is seen as acceptable when prioritizing alleviation of suffering, respect for autonomy, and providing a dignified death (patient autonomy, divine based non- maleficence).
Treatment withholding/withdrawal			
Muishout, G., Topcu, N., de la Croix, A., Wiegers, G., & van Laarhoven, H. W. (2022). (prior experienced tension)	Second opinion requirement in majors	Divine based non-maleficence (family) versus non-maleficence and/or patient autonomy (caregivers; APB Turkish mams)	Muslims faced with a proposal to withdraw treatment for a family member, after all options have been exhausted, may feel religiously obligated to oppose it and seek a second opinion due to concerns about afterlife consequences (divine based non-maleficence). This may exclude medical-ethical considerations, such as futile care and patient dignity, and hinder informed decision-making (patient autonomy).
Oosterveld-Vlug, M. G., Francke, A. L., Pasman, H. R. W., & Onwuteaka-Philipsen, B. D. (2017). (potential future tension)	Faith based rejection of withdrawal rationale	Divine based beneficence (family) versus non-maleficence (caregivers; APB Muslim participants)	Medical rationales for treatment withdrawal, such as curative overtreatment or life prolongation (non-maleficence), may be perceived by Muslim patients and families as religiously inappropriate due to the duty to seek healing (divine based beneficence), potentially complicating clinical decision-making and communication.
Suleman M. (2023). (prior experienced tension)	Faith-based hope versus medical proportionality	Divine based beneficence (family; vs. non-maleficence (caregivers; APB care providers)	Religion-based hope for a cure (grounded in divine based beneficence) may lead to requests for medical interventions that are perceived by clinicians as ambiguous or inappropriate in end-of-life care, potentially complicating decisions guided by non-maleficence clinical proportionality (non-maleficence).
Kolmar, A., Kamal, A. H., & Steinhauser, K. E. (2022). (prior experienced tension)	End-of-life decisions in minors	Divine based non-maleficence (family; vs. non-maleficence (caregivers; APB care providers)	The belief among Muslims that death is determined by God’s makes it challenging to discuss cessation of life-sustaining therapies for minor patients (non-maleficence).
Lundqvist, A., Nilstun, T., & Dykes, A. K. (2003). (potential future tension)	Withdrawing treatment in neonatal care	Divine based non-maleficence (family) versus non-maleficence (caregivers; APB Muslim women)	Withdrawing treatment in neonatal care to prevent suffering (non-maleficence) conflicts with religious values that affirm God’s control over life and death (divine based non maleficence), leaving no room for discussion or shared decision-making.

## End of life communication

In four studies, the tension between Divine based Non-maleficence (family) and Patient Autonomy (caregivers) centered on open communication with patients about impending death.^26,28,31,33^ Two studies underscored the real tension experienced by care providers who felt that withholding information from the dying—as requested by family and friends—compromised transparency and patient autonomy, thus affecting the quality of life and the dying process.^31,33^ While the first one involved local care providers in a European country, the second one involved non-Muslim expat nurses in a Muslim-majority country.^31,33^ The other two studies explored potential future tensions as expected by potential patients/relatives.^26,28^ The first study highlighted a preference for realistic end-of-life communication with family members rather than the patient. Muslim participants suggested that physicians should inform the patient and/or relatives about the lack of curative treatments but avoid stating the illness is incurable, as life and death decisions are seen as within God’s sole territory.^26^ The fourth study described a paradox where Arabs (both Christians and Muslims) in an attempt to protect patients, suggested care providers should avoid terms like ‘death’ and ‘cancer’, since patients would not be able to handle the truth, and give up hope for healing, while this was solely in God’s power. However, these same participants expressed a desire for full disclosure if they were dying themselves, to reconcile with family and friends in preparation for the afterlife.^28^

## Spiritualizing pain

In four studies, the tension between Non-maleficence vs. Divine-based Beneficence emerged in the context of pain relief during end-of-life care.^23,25,30,34^ Two studies, using the same data set, described the tension experienced by Muslim doctors in Euro-American contexts. These doctors faced a conflict between their professional ethical duty to relieve a dying patient’s pain and their religious duty and the family’s religious desire to let Muslim patients experience pain as a means of seeking forgiveness for sins in preparation for the afterlife.^23,25^ The third study explored a hypothetical tension between non-maleficence, patient autonomy, and divine based beneficence in an end-of-life care scenario. A male Muslim participant stated he would refuse pain relief that might unintentionally hasten death, preferring to endure pain for spiritual reasons.^30^ This contrasted with other participants who favored pain management.^30^ The fourth study involved a questionnaire among final-year Muslim medical students in a Muslim-majority country, highlighting the hypothetical tension between divine-based non-maleficence and patient autonomy, given that euthanasia is forbidden in the country.^34^ The former principle included the religious prohibition against intentionally killing a patient (the majority view among respondents) versus the patient’s right to a self-determined and therefore dignified death.^34^

## Treatment withholding/withdrawal

In five studies, tension arose from conflicting divine based Beneficence or Non-maleficence as understood by families, versus non-divine approaches to the principle of Non-maleficence as upheld by caregivers in the context of treatment withdrawal for both adults and minors.^24,26,27,29,32^ One study highlighted actual experienced tension arising from the perception and standard advice of participating imams in a non-Muslim majority country. These imams advised family members of critically or terminally ill patients to initially refuse any proposals to withdraw treatment, seek second opinions, and continue searching for a cure. This advice was driven by concerns about the afterlife consequences for all Muslims involved (both the imam and family members) who agreed with such proposals.^24^ A second study identified tensions perceived by care providers in an Euro-American country, stemming from the belief that Muslims (and religious people in general) are culturally and religiously opposed to treatment restrictions in minors, as they believe God decides matters of life and death. This belief was supposed to lead a priori to conflicts with family members refusing to discontinue treatment.^29^ A third study highlighted actually experienced and hypothetical tension perceived by Muslim mothers in an Euro-American country regarding the withdrawal of treatment for neonates, including data from both mothers with actual experience and those without. These mothers, because of their belief that God should decide matters of life and death, indicated their unwillingness to engage in any discussions about treatment withdrawal.^32^ A fourth study described how religion-based hope for a cure among Muslim patients and families may lead to requests for medical interventions that, from a medical perspective, might be seen as ambiguous or even inappropriate in the context of end-of-life care.^27^ Finally, a fifth study emphasized that curative overtreatment and life prolongation, as rationales for continuing or withdrawing treatment, are considered inappropriate concepts for Muslims due to the religious imperative to seek healing, indicating potential future tension in similar contexts.^26^

## Discussion

This study explored how the religio-culturally grounded values of Muslim patients and their families regarding end-of-life care may conflict with biomedical ethical principles in Euro-American healthcare settings, leading to tensions arising from differing interpretations or prioritizations of ethical principles.

In the first theme ‘*End of life communication’* the main tension concerned Muslim families’ desire to control the information conveyed to the dying patient aimed at protecting the patient from losing hope for healing versus healthcare providers’ emphasis on transparency and respect for patient autonomy. Revealing in this context is the paradox of wanting to know one’s own prognosis in a hypothetical end-of-life scenario, particularly for religiously inspired preparation for the afterlife, while at the same time being hesitant to disclose such information to someone else in a similar situation, out of fear that it would be too mentally burdensome. This raises the question whether such nondisclosure is primarily guided by a protective concern for the patient’s emotional resilience, or whether it also may reflect an attempt to shield oneself from the psychological burden of openly confronting the patient’s condition.^28,35,36^ Studies into patient views about truth-telling from Muslim-majority countries confirm this pattern, revealing a gap between individuals’ wish for full disclosure in the event of their own illness and the prevailing social practice of information veiling for others.^37–42^ Additionally, a small body of literature from doctors’ perspectives in these regions shows growing support for acknowledging patients’ wishes and rights to full disclosure.^43–45^ Complementing these findings, Islamic e- fatāwās—religious rulings by Islamic scholars to guide ethical decisions—offer a religious rationale for non-disclosure, emphasizing divine knowledge of death and the importance of preserving hope, though one fatāwā affirms the patient’s basic right to know, provided they are mentally capable of handling this information.^46–49^ A study on the experiences of expatriate nurses in Qatar on the other hand, illustrates how the impact of the religiously grounded principle of non-maleficence in the context of cancer non-disclosure, can inadvertently cause multifaceted harm, including undermining the Islamic obligation for patients to seek reconciliation before death.^50^ While there is considerable variation by country in the Middle East, as well as by patients’ age, concerning opinions on truth-telling, the growing preference for full disclosure among patients and healthcare professionals may be interpreted as part of a gradual and context-dependent shift toward greater openness. This trend appears to be influenced by factors such as globalization, increased public education, and evolving cultural attitudes toward discussing death and dying.^51,52^ Interestingly, one study specifically found that Jordanian doctors who consistently practiced truthfulness were more likely to view non-disclosure as unethical, highlighting a nuanced relationship between their commitment to honesty and their ethical perspectives as medical professionals.^44^ This tension between protecting the patient’s emotional state and respecting their right to know calls for a reflection on informed consent, a foundational principle in the dominant Euro and North American healthcare paradigm. Without the patient’s informed agreement, any medical intervention risks violating their autonomy and moral integrity. A deontological perspective frames truth-telling as a non-negotiable duty, while consequentialist reasoning may justify non-disclosure to prevent harm or preserve hope.^53^ In various cultural contexts outside the dominant Euro-American healthcare paradigm, such reasoning is often rooted in paternalistic traditions, where beneficence plays a central role in clinical decision-making. However, as argued throughout this section, religio-cultural norms are not fixed.^54^

Reconsidering informed consent in this ethically plural landscape invites a more relational and patient-centered approach- one that does not reduce care to a choice between deontological duty and consequentialist outcome, but instead seeks to understand how truth-telling is shaped by cultural, familial, and religiously grounded conceptions of care. Religio-cultural norms—such as divine-based, family-centered decision-making—are intersectional and dynamic. These norms embody ethical reasoning grounded in religious obligations to God and fellow humans, typically mediated through family-based decision-making structures (see Table 3). While such frameworks may initially support non-disclosure, they may also evolve toward a deontological emphasis on the patient’s right to know, particularly when religious obligations—such as preparing for death—are foregrounded. This underscores the importance of viewing religio-cultural norms not as static barriers to informed consent, but as potential ethical resources that can align with, and even reinforce, the patient’s moral agency. Such an approach opens space for clinicians to engage with patients as moral agents, navigating disclosure through dialog that is ethically attentive and responsive to the diverse frameworks that inform patients’ expectations and needs at the end of life.

In the second theme ‘*Spiritualizing pain’*, the main tension concerned the ethical duty of caregivers to alleviate pain versus the religious desire of patients and family members to endure pain for religious purposes. The argument to endure suffering through pain can be situated within the broader theological discourse on reconciling an omnipotent God with the existence of evil and suffering (theodicy). In Islamic theology, illness may be interpreted as a manifestation of Divine mercy, through which sins are expiated and the individual is elevated to a higher spiritual state.^56,57^ Within this framework, the refusal of pain relief in end-of-life care can thus be understood as an active, instrumental choice, motivated by an eschatological orientation, whereby the patient or their family elects to endure, or permit the endurance of, pain as a means of spiritual purification and preparation for the afterlife.^55–57^ While this orientation may be meaningful to patients and families, it can come into tension with the framing of pain relief as a human right, which emphasizes the obligation to prevent avoidable suffering.^58^ In cases where patients are no longer able to express their wishes, surrogate decisions to permit suffering may raise ethical concerns about dignity, autonomy, and the right to adequate pain management, placing a considerable moral burden on care providers who must navigate between religious sensitivities and their professional duty to alleviate suffering. Hesitancy among some Muslims regarding the use of pain relief at the end of life may also stem from a conceptual misunderstanding, shared by both Muslim healthcare providers and receivers, in which the proportionate administration of morphine or the application of palliative sedation is erroneously perceived as hastening death, an outcome that would render its use religiously impermissible.^57,59,60^ These theological concerns are addressed through fatāwā—religious-ethical opinions issued by qualified Islamic scholars in response to specific questions from individuals, families, or institutions. A fatāwā is not a binding legal verdict, but a form of applied religious reasoning that draws on Islamic scripture, tradition, and scholarly interpretation to guide moral decision-making.^61^ In contemporary settings, many such opinions are published online as e-fatāwā, making them accessible to Muslims across diverse cultural and national contexts.^62,63^ As culturally embedded outputs of applied theology, fatāwā offer insight into how jurists negotiate scriptural principles with clinical realities. Although shaped by context and not always representative of broader consensus, they serve as discursive mirrors of Muslim moral reasoning at the end of life, including the permissibility of pain relief and sedation.^64^ Yet despite the growing clinical relevance of these issues, only a handful of fatāwā explicitly address palliative sedation. The following examples illustrate how applied theology may align—or come into tension—with medical standards concerning pain management at the end of life. One such example is a fatwa by Ibn Baz, the late Grand Mufti of Saudi Arabia, who affirmed that the use of sedatives to relieve the pain of the dying, even to the extent that the patient loses consciousness, is permissible, provided that it facilitates the departure of the soul and does not hasten death. This ruling highlights a nuanced position within Islamic jurisprudence that legitimizes compassionate pain management, including palliative sedation while maintaining theological and ethical boundaries.^64^ Similarly, a fatāwā issued by the Saudi-based Islamic Fiqh Academy (IFA) emphasizes the medical obligation to alleviate pain in end-of-life care in general.^65^ Yet, this position may come into tension with a dying Muslim patient’s desire to remain conscious in order to fulfill religious duties, such as performing the five daily prayers and reciting the Islamic testimony of faith (shahada) in their final moments.^50^ In contrast, another e-fatāwā argues that palliative sedation is generally impermissible. It reasons that the soul’s departure is a metaphysical process unaffected by painkillers; that sedation may prevent the dying from reciting the shahada; that no compelling medical or religious justification exists; and that the uncertain timing of death makes unnecessary sedation potentially harmful.^66^ To date, only a few publicly available fatāwā address palliative sedation, forming a narrow but significant spectrum within Islamic jurisprudence, from conditional permissibility to general prohibition. This scarcity highlights an underdeveloped area of Islamic legal thought, despite the growing relevance of end-of-life care. The lack of clear theological guidance may contribute to uncertainty among Muslim patients and families, reinforcing concerns about missing prayers or the shahada due to sedation. As a result, even medically justified sedation may be met with reluctance, underscoring the need for more fatāwās and engagement from religious scholars.^57^

The third theme *‘Treatment Withholding & Withdrawal’* underscores the tension between the care providers perspective on treatment restrictions and avoiding harm as opposed to the religiously driven high regard for preserving human life among Muslims, which may be an incentive for the pursuit of life-prolonging and/or ultimate lifesaving therapeutic interventions in end-of-life settings.^13,66^ Nevertheless, this primary commitment is subject to normative constraints, as Islamic jurisprudence (predominantly) grants doctors the authority to withhold or withdraw treatment when death is deemed inevitable and the anticipated harm, including *spiritualizing pain*, outweighs the potential therapeutic goal of prolonging life.^13,65,67,68^ A complicating factor is the normatively endorsed belief in the possibility of miracles among some Muslims in end-of-life care decision-making.^12,65^ This belief may lead patients or their family members to resist proposed clinical decisions to withhold or withdraw treatment, viewing such decisions as prematurely closing the door to a miraculous recovery.^12^

## What the study adds and implications for practice

This review makes a novel contribution to end-of-life care for Muslim patients in Western Euro-American healthcare settings by systematically mapping key ethical tensions through a structured ethical framework, while integrating theological insights with empirical findings to offer a more comprehensive ethical analysis. Our findings highlight the complex role of religion, both as lived religion and as applied (normative) theology, in shaping end-of-life care dynamics for Muslim patients and their families within Euro-American healthcare settings. These religio-cultural dimensions may introduce ethical tensions, particularly when differing frameworks prioritize distinct moral principles. By examining the lived realities of Muslim patients and families, and later contextualizing these through Islamic legal opinions, we show that Islamic perspectives on end-of-life care are not monolithic. Rather, they encompass a range of interpretations that can offer meaningful opportunities for navigating ethical divergences in practice. For example, the plurality of views found in contemporary e-fatāwās on pain management reflects interpretive flexibility. This diversity can be leveraged to foster a productive *intermediary space* that bridges ethical and cultural divides in end-of-life care. Muslim chaplains can play a key role in this process by mediating between perspectives and offering *cultural subtitles* that foster mutual understanding.^9^ Attention should also be given to intergenerational dynamics within Muslim families. Cultural taboos around open communication may lead younger family members to assume that elders prefer aggressive life-prolonging treatment, when in fact they may favor comfort-focused care that prioritizes quality of life.^69^ Diaspora contexts may generate distinct dynamics in how Muslim families conceptualize good care at the end of life. First-generation migrants may carry religio-cultural care expectations rooted in the country of origin, while second- and third-generation individuals may reinterpret these norms through evolving cultural and religious identities.^71^ This intergenerational divergence can shape differing views on autonomy, suffering, and appropriate communication. Moreover, Muslim communities in diaspora are internally diverse, originating from different countries with varying healthcare systems and culturally embedded notions of appropriate end-of-life care. This diversity further complicates assumptions about shared values and highlights the importance of nuanced, individualized engagement, and underscores the need for context-sensitive research into how evolving religio-cultural identities shape ethical attitudes in end-of-life care.

However, studies about end of life care perception and second-, third-, and later generations of Muslims in non-Muslim majority societies are underexplored.

An area warranting further exploration is the presence of stigma and religio-culturally shaped misconceptions among Muslims from Muslim majority countries regarding palliative care and opioid-based pain management, which may be reproduced in Euro-American healthcare contexts. In patient and family interactions, addressing this gap requires focused dialog and education at the patient and family level.^57^ At the meso level, broader engagement is needed, ideally in collaboration with Muslim grassroots or umbrella organizations (both national and transnational), local mosques, and imams, to develop a shared (conceptual) framework for end-of-life care. Such a framework would enable healthcare providers and Muslim patients to navigate care decisions based on a common conceptual understanding. This could involve mosque-based training initiatives aimed at increasing awareness among Muslim communities about Islamic normative perspectives on end-of-life care and the range of ethically permissible options in clinical settings^70^

While this review identifies key ethical tensions in end-of-life care for Muslim patients, it also reveals a notable gap in the empirical literature regarding how clinicians navigate these tensions in practice. Few studies offer insight into the strategies, adaptations, or decision-making processes employed by healthcare providers when confronted with religio-cultural tensions. This absence limits our understanding of how ethical principles are operationalized in real-world clinical settings and underscores the need for future research focused on clinical responses to these tensions.

These findings also carry important policy implications. In particular, patient-provider communication around end-of-life pain alleviation must account for religious motivations to endure pain, which are rooted in Islamic theological views on suffering and redemption. Policies should support culturally sensitive dialog and facilitate religiously informed decision-making, particularly in areas where Islamic jurisprudence offers limited guidance—such as the permissibility of palliative sedation. While euthanasia is legally permitted in several Euro-American healthcare systems (e.g. the Netherlands, Belgium, Luxembourg, Spain, Canada, and parts of Australia), tensions may arise when Muslim patients autonomously opt for it while family members object on religious grounds. Policy frameworks should anticipate such conflicts and offer pathways for respectful dialog, Muslim chaplain involvement, and ethically acceptable alternatives such as palliative sedation. Overall, policy frameworks should promote ethical pluralism and individualized care that aligns with both biomedical and Islamic ethical principles.

## Strengths and limitations

A key strength of this review lies in its novel contribution of systematically mapping ethical tensions through a structured ethical framework, while integrating both theological and empirical perspectives to enrich the analysis. Another key strength of this study is the multidisciplinary composition of the research team, which brought together expertise in systematic review methodology and search strategy (JCFC), Islamic ethics (GM, SEA), religious studies (GM, LP), and clinical oncology and palliative care (LVZ, SEA, LP). This diverse expertise informed the interpretation of findings within the broader discussion. One limitation is the exclusion of gray literature and the restriction to English-language publications. As a result, relevant research published in languages spoken in Muslim-majority countries, such as Arabic, Indonesian, Malay, Turkish, and Urdu, was not included. Despite this limitation, the study’s focused approach offers valuable insights into the religious and cultural dimensions that shape ethical dilemmas in the clinical care of Muslim patients and their families. Moreover, the findings highlight areas in need of further development to support a shared understanding of end-of-life care, one that is both acceptable within the religio-cultural framework of Muslim communities and compatible with Euro-American clinical practice. In doing so, this study contributes to the ongoing search for common ground in ethically and culturally sensitive care.

## Conclusion

This study aimed to map religio-cultural ethical issues that give rise to tensions in end-of-life care for Muslim patients within Euro-American healthcare settings. Three principal themes were identified where religio-cultural ethical differences contribute to such tensions: *(1) end-of-life communication; (2) spiritualizing pain*; and *(3) treatment withholding and withdrawal.* For each theme, the complex role of religion, both as lived experience and as normative theological interpretation, was examined. The findings demonstrate that Islamic perspectives on end-of-life care are not monolithic; rather, they encompass a spectrum of interpretations that may offer doctors, Muslim patients, and their families meaningful opportunities to navigate ethical divergences in clinical practice. The current body of research reveals a lack of guidance for healthcare providers on how to identify and address these tensions early in the care process. To counter religio-culturally informed misconceptions, particularly those related to communication about palliative care and the use of opioid-based pain management, we propose the development of a shared conceptual framework for end-of-life care. This should be accompanied by the creation of constructive intermediary spaces that bridge ethical and cultural divides. The successful implementation of these initiatives requires the active involvement not only of healthcare providers, patients, and families, but also of Muslim umbrella organizations, mosques, imams, and Muslim chaplains or spiritual care providers. Together, these stakeholders can contribute to a more ethically and culturally responsive model of end-of-life care.

## Supplemental Material

sj-docx-1-pmj-10.1177_02692163251407877 – Supplemental material for Mapping potential cultural and religious tensions in end-of-life care for Muslim patients: A scoping review Supplemental material, sj-docx-1-pmj-10.1177_02692163251407877 for Mapping potential cultural and religious tensions in end-of-life care for Muslim patients: A scoping review by George Muishout, Lenneke Post, Salima El Ayachi, Johannes C. F. Ket and Lia van Zuylen in Palliative Medicine
