# Supplementary material for: Mapping potential cultural and religious tensions in end-of-life care for Muslim patients: A scoping review
Source: Palliat Med. 2026 Jan 21;40(4):432–47. doi: 10.1177/02692163251407877 (PMC13062463; doi:10.1177/02692163251407877)
Supplement: sj-docx-1-pmj-10.1177_02692163251407877 – Supplemental material for Mapping potential cultural and religious tensions in end-of-life care for Muslim patients: A scoping review [file sj-docx-1-pmj-10.1177_02692163251407877.docx]

**Post L et al Supplementary Information: full search strategies**

**Johannes C.F. Ket**

**Search strategy for Elsevier/Scopus (7 November 2024)**

| **3** | #1 AND #2 | **5,923** |
| --- | --- | --- |
| **2** | TITLE-ABS-KEY (palliati* OR end-of-life OR (terminal* W/3 ill*) OR death* OR dying OR die OR decease* OR euthan* OR life-threat* OR mercy-killing* OR physician-assisted-suicid* OR hospice* OR (truth W/2 tell*) OR ((withdraw* OR withhold* OR with-draw* OR with-hold* OR withheld OR with-held OR discont*) W/3 (treat* OR therap*)) OR incurab* OR noncurab*) | **3,092,464** |
| **1** | TITLE-ABS-KEY (islam* OR wahhabis* OR wah-habis* OR salafis* OR soenni* OR sunni* OR shi-is* OR shi-a OR sji-is* OR quran* OR muslim* OR sufis* OR soefis* OR imam* OR muhammad*) | **161,671** |

**Search strategy for OVID/Medline (7 November 2024)**

| **1** | "Islam"/ or (islam* or wahhabis* or wah-habis* or salafis* or soenni* or sunni* or shi-is* or shi-a or sji-is* or quran* or muslim* or sufis* or soefis* or imam* or muhammad*).ti,ab,kf. | **19,277** |
| --- | --- | --- |
| **2** | exp "Palliative Medicine"/ or exp "Palliative Care"/ or exp "Terminal Care"/ or "Death"/ or exp "Euthanasia"/ or exp "Hospice Care"/ or exp "Hospices"/ or exp "Hospice and Palliative Care Nursing"/ or "Withholding Treatment"/ or exp "Terminally Ill"/ or (palliati* or end-of-life or (terminal* adj3 ill*) or death* or dying or die or decease* or euthan* or life-threat* or mercy-killing* or physician-assisted-suicid* or hospice* or (truth adj2 tell*) or ((withdraw* or withhold* or with-draw* or with-hold* or withheld or with-held or discont*) adj3 (treat* or therap*)) or incurab* or noncurab*).ti,ab,kf. | **1,559,607** |
| **3** | 1 and 2 | **1,633** |

**Search strategy for Ebsco/CINAHL (7 November 2024)**

| **#** | **Query** | **Results** |
| --- | --- | --- |
| **S3** | S1 AND S2 | **915** |
| **S2** | MH "Palliative Care" OR MH "Palliative Care Nursing" OR MH "Palliative Medicine" OR MH "Terminal Care" OR MH "Terminally Ill Patients+" OR MH "Death" OR MH "Euthanasia" OR MH "Hospice Care" OR MH "Suicide, Assisted" OR MH "Euthanasia, Passive" OR TI(palliati* OR end-of-life OR (terminal* N3 ill*) OR death* OR dying OR die OR decease* OR euthan* OR life-threat* OR mercy-killing* OR physician-assisted-suicid* OR hospice* OR (truth N2 tell*) OR ((withdraw* OR withhold* OR with-draw* OR with-hold* OR withheld OR with-held OR discont*) N3 (treat* OR therap*)) OR incurab* OR noncurab*) OR AB(palliati* OR end-of-life OR (terminal* N3 ill*) OR death* OR dying OR die OR decease* OR euthan* OR life-threat* OR mercy-killing* OR physician-assisted-suicid* OR hospice* OR (truth N2 tell*) OR ((withdraw* OR withhold* OR with-draw* OR with-hold* OR withheld OR with-held OR discont*) N3 (treat* OR therap*)) OR incurab* OR noncurab*) OR SU(palliati* OR end-of-life OR (terminal* N3 ill*) OR death* OR dying OR die OR decease* OR euthan* OR life-threat* OR mercy-killing* OR physician-assisted-suicid* OR hospice* OR (truth N2 tell*) OR ((withdraw* OR withhold* OR with-draw* OR with-hold* OR withheld OR with-held OR discont*) N3 (treat* OR therap*)) OR incurab* OR noncurab*) | **417,857** |
| **S1** | MH "Islam" OR MH "Muslims" OR MH "Quran" OR TI(islam* OR wahhabis* OR wah-habis* OR salafis* OR soenni* OR sunni* OR shi-is* OR shi-a OR sji-is* OR quran* OR muslim* OR sufis* OR soefis* OR imam* OR muhammad*) OR AB(islam* OR wahhabis* OR wah-habis* OR salafis* OR soenni* OR sunni* OR shi-is* OR shi-a OR sji-is* OR quran* OR muslim* OR sufis* OR soefis* OR imam* OR muhammad*) OR SU(islam* OR wahhabis* OR wah-habis* OR salafis* OR soenni* OR sunni* OR shi-is* OR shi-a OR sji-is* OR quran* OR muslim* OR sufis* OR soefis* OR imam* OR muhammad*) | **9,595** |

**Search strategy for Ebsco/APA PsycINFO (7 November 2024)**

| **#** | **Query** | **Results** |
| --- | --- | --- |
| **S3** | S1 AND S2 | **766** |
| **S2** | DE "Hospice" OR DE "Palliative Care" OR DE "Assisted Suicide" OR DE "Death Attitudes" OR DE "Death Rites" OR DE "Palliative Care" OR DE "Terminally Ill Patients" OR TI(palliati* OR end-of-life OR (terminal* N3 ill*) OR death* OR dying OR die OR decease* OR euthan* OR life-threat* OR mercy-killing* OR physician-assisted-suicid* OR hospice* OR (truth N2 tell*) OR ((withdraw* OR withhold* OR with-draw* OR with-hold* OR withheld OR with-held OR discont*) N3 (treat* OR therap*)) OR incurab* OR noncurab*) OR AB(palliati* OR end-of-life OR (terminal* N3 ill*) OR death* OR dying OR die OR decease* OR euthan* OR life-threat* OR mercy-killing* OR physician-assisted-suicid* OR hospice* OR (truth N2 tell*) OR ((withdraw* OR withhold* OR with-draw* OR with-hold* OR withheld OR with-held OR discont*) N3 (treat* OR therap*)) OR incurab* OR noncurab*) OR KW(palliati* OR end-of-life OR (terminal* N3 ill*) OR death* OR dying OR die OR decease* OR euthan* OR life-threat* OR mercy-killing* OR physician-assisted-suicid* OR hospice* OR (truth N2 tell*) OR ((withdraw* OR withhold* OR with-draw* OR with-hold* OR withheld OR with-held OR discont*) N3 (treat* OR therap*)) OR incurab* OR noncurab*) | **192,900** |
| **S1** | DE "Islam" OR DE "Muslims" OR TI(islam* OR wahhabis* OR wah-habis* OR salafis* OR soenni* OR sunni* OR shi-is* OR shi-a OR sji-is* OR quran* OR muslim* OR sufis* OR soefis* OR imam* OR muhammad*) OR AB(islam* OR wahhabis* OR wah-habis* OR salafis* OR soenni* OR sunni* OR shi-is* OR shi-a OR sji-is* OR quran* OR muslim* OR sufis* OR soefis* OR imam* OR muhammad*) OR KW(islam* OR wahhabis* OR wah-habis* OR salafis* OR soenni* OR sunni* OR shi-is* OR shi-a OR sji-is* OR quran* OR muslim* OR sufis* OR soefis* OR imam* OR muhammad*) | **14,696** |
